# Candidemia in the Dominican Republic: species distribution, resistance, clinical characteristics, and outcomes at a tertiary care hospital

**DOI:** 10.1017/S0950268825100496

**Published:** 2025-08-26

**Authors:** Rita Rojas-Fermín, Javier Rojas-Jiménez, Marlon Rojas-Jimenez, Anel Guzmán-Marte, Ann Sánchez, Alfredo J. Mena Lora

**Affiliations:** 1 Departamento de Infectologia, Hospital General Plaza de la Salud, Santo Domingo, Dominican Republic; 2 Escuela de medicina, Universidad Nacional Pedro Henríquez Ureña, Santo Domingo, Republica Dominicana; 3 Division of Infectious Diseases, Department of Medicine, University of Illinois at Chicago, Chicago, IL, USA

**Keywords:** Candidemia, antifungals, antimicrobial resistance, Global Health, Fungal infections

## Abstract

Bloodstream infections (BSIs) caused by *Candida* are a significant cause of morbidity and mortality. Geographical variations exist in the epidemiology of candidemia, with a paucity of data in the many low- and middle-income countries. We performed a retrospective study of candidemia from 2017 to 2022 at a 289-bed teaching hospital in the Dominican Republic (DR). A total of 197 cases were reviewed. Overall mortality rate was 49.2%. Age and vasopressor use were associated with mortality. The most prevalent *Candida* species were *C. tropicalis* and *C. parapsilosis. C. albicans* was 12% resistance to amphotericin B. These findings underscore the importance of understanding local epidemiology and may help inform empiric therapy and the development of treatment guidelines in the DR.

## Background

Bloodstream infections (BSI) caused by *Candida* are a significant cause of morbidity, often reported among the top five most common causes of hospital-acquired BSIs [1]. Despite advancements in medical care, candidemia-related mortality rates remain high. Mortality from candidemia has been reported as high as 47%, and this risk increases for those with septic shock [1, 2]. Early empiric antifungal therapy is key to improve patient outcomes [3]. However, the emergence and spread of antifungal resistance complicates management and poses a major public health threat [4]. Geographical variations exist in the epidemiology of candidemia, with a paucity of data currently available about the disease’s epidemiology and resistance patterns in the many low- and middle-income countries (LMIC) [5, 6]. Understanding local epidemiology and susceptibility patterns is key to help guide empiric therapy. Our aim is to describe the epidemiology of candidemia at a tertiary care hospital in the Dominican Republic (DR) and provide local insights that may inform clinical care and stewardship efforts.

## Methods

### Study setting and design

This is a retrospective study conducted at a tertiary hospital in the DR. The facility is a 289-bed teaching hospital that serves medical, surgical, and solid organ transplant patients. A comprehensive review of electronic medical records was performed to extract data regarding candidemia cases from 1 January 2017 to 31 December 2022. Clinical and demographic variables, including patient age, gender, and underlying co-morbidities, were collected along with microbiology data, such as *Candida* species, and antifungal resistance patterns.

### Microbiology

Blood cultures were incubated using Bact-ALERT (BioMérieux) automated system. Yeast isolates were identified, and susceptibility testing was performed in the Vitek 2 compact (bioMerieux, France), using the Clinical and Laboratory Standards Institute breakpoints (CLSI M27M44S). [7]

### Inclusion and exclusion criteria

The inclusion criteria consisted of all patients within the specified study period who had a laboratory-confirmed diagnosis of candidemia. Candidemia was defined as the isolation of *Candida* species from one or more blood cultures in a patient exhibiting clinical signs of infection. If multiple blood cultures were positive, a new episode was defined only if more than 30 days had passed since the initial positive blood culture. Patients with incomplete medical records or those lacking confirmation of candidemia were excluded from the analysis.

### Data analysis

Descriptive statistics were employed to summarize the clinical and demographic characteristics of the candidemia cases. Categorical variables were compared using chi-square or Fisher’s exact tests and continuous variables using Student’s *t*-test. Univariate associations between comorbidities, demographic factors, species, and mortality were assessed using crude odds ratios. To identify independent predictors of mortality, we performed multivariable logistic regression analyses. Variables with a *p*-value <0.2 in univariate testing were considered for inclusion. Demographic variables such as age, sex, and comorbidities with sufficient representation (≥5 cases in both survivor and non-survivor groups) were included. Odds ratios (ORs), 95% confidence intervals (CIs), and *p*-values were reported, with significance set at *p* < 0.05.

The distribution of *Candida* species and their antifungal resistance patterns was analysed. For the evaluation of the susceptibility profile of the *Candida* isolates, the percentage of sensitivity of the isolates according to the established minimum inhibitory concentration cut-off points. Patient outcomes, including mortality rates and length of hospital stay, were assessed to provide valuable insights into the clinical impact of candidemia within the study population.

### Ethics

The institutional review board at the Hospital General Plaza de la Salud reviewed and approved this study.

## Results

A total of 210 samples were collected from 197 patients, of which 103 (52.28%) were men and the average age was 53 (IQR 54). Age distribution was 26 (13%) patients <1 year of age, 21 (10%) were 1–10 years, 6 (3%) 11–17 years, 22 (11%) 18–35 years, 46 (23%) 36–59 years, and 76 (38%) were over 60 years of age (Table 1).

**Table 1.** Age distribution of patients with candidemia

Age groups	Frequency (*n*)	Percent (%)
<1	26	13.19
1–10	21	10.66
11–17	6	3.04
18–35	22	11.16
36–59	46	23.35
>60	76	38.57

### Demographic, clinical characteristics, and outcomes

**Figure 1.**
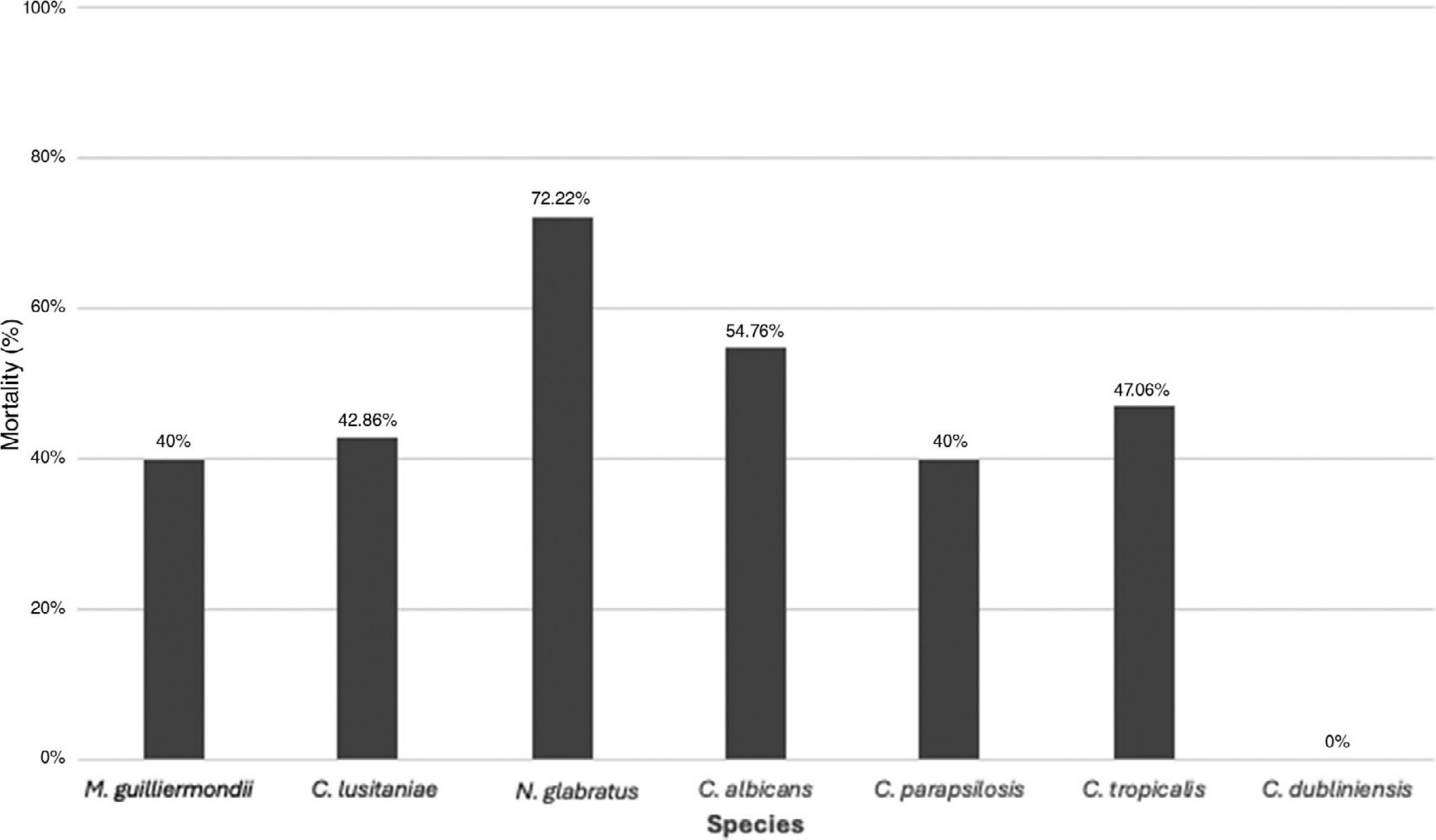
Distribution of *Candida* species by mortality rates (%).

Stays in the intensive care unit (ICU) (74.1%) and prior antibiotic exposure (92.9%) were the most prevalent clinical characteristics, followed by prolonged length of stay, vasopressor use, and the presence of central venous catheters. Median LOS was 19 days for all patients and 26 days for surviving patients. The overall mortality rate for all patients was 49.2%. Mortality was highest for patients with spinal diseases (57.14%), cerebrovascular disease (55.81%), malignancy (43.48%), cardiomyopathy (42.11%), auto-immune disorders (38.46), diabetes (39%), hypertension (35.54%), and SARS-CoV-2 (32%) (Table 2). Mortality varied by causal species, with the highest mortality for *Nakaseomyces glabratus* (72.2%), followed by *C. albicans* (54.76%), *C. tropicalis* (47.06%), *Clavispora lusitaniae* (42.86%), *C. parapsilopsis* (40%), and *Meyerozyma guiliermondii* (40%) (Figure 1).

**Table 2.** Comorbidities of patients with candidemia by survival

Comorbidity	Mortality (*N*)	Mortality (%)	*X*2	*P* value
Pulmonary condition	42	46.67	1.112	0.292
Hypertension	38	36.54	17.830	<0.001*
Cerebrovascular disease	24	55.81	0.562	0.454
Diabetes mellitus	21	39.62	3.599	0.058
Malignancy	20	43.48	1.274	0.259
Nephropathy	18	28.57	18.246	<0.001*
Cardiomyopathy	16	42.11	1.411	0.235
Hepatic condition	8	26.67	8.220	0.004*
SARS-COV–2 infection	8	32	4.032	0.045
Autoimmune disease	5	38.46	0.842	0.359
Spinal diseases	4	57.14	0.118	0.731

*Note:* * = “Statistically significant”

### Univariate and multivariate analysis

Among 197 patients with candidemia, the overall mortality rate was 49.2%. Univariate analysis identified variables associated with mortality, including age, ICU admission, vasopressor use, mechanical ventilation, and several comorbidities (Supplement 1–3). *Candida* species did not show statistically significant differences in mortality in unadjusted analysis. Vasopressor use showed a strong association with mortality (crude OR ≈ 2.5). Vasopressor use remained an independent predictor of mortality in multivariate analysis (adjusted OR 2.49; 95% CI, 1.23–4.93; *p* = 0.009). Mechanical ventilation and transplant status showed trends towards association but were not statistically significant (Supplement 1–3). Age was independently associated with mortality (adjusted OR 1.02 per year; 95% CI, 1.007–1.027; *p* = 0.001). Sex and individual comorbidities did not remain significant in the adjusted model, potentially due to sample size limitations or collinearity (Supplement 1–3).

### Microbiology and fungal resistance

The most common species was *C. tropicalis* (*n* = 90, 43%), followed by *C. parapsilosis* (*n* = 50, 24%), *C. albicans* (*n* = 42, 20%), *N glabratus* (*n* = 18, 8.5%), *C. lusitaniae* (*n* = 4, 2%), *MM. guillermondii* (*n* = 2, 1%), *Trichomonascus ciferrii* (*n* = 2, 1%), and *C. dublinensis* (*n* = 1, 0.5%). No cases of *Candidozyma auris* or *Pichia pichiakudriavzevii* species were isolated during the study period. *C. albicans* was 12% resistant to amphotericin B and 5% resistant to fluconazole (Table 3). *N. glabratus* was 11% resistant to amphotericin B, 5.5% resistant to fluconazole. *Clavispora lusitaniae* exhibited 14% resistance to amphotericin B. *C. parapsilosis* exhibited 12% resistant to fluconazole, and 2% resistance to voriconazole. *C. tropicalis* was 4% resistant to fluconazole. *C. dublinensis*, *T. ciferrii*, and *M. guillermondii* exhibited no resistance. No resistance to echinocandins was found across all species.

**Table 3.** *Candida* species antifungal susceptibilities (%)

Species	AmpB resistant (%)	Fluconazole intermediate (%)	Fluconazole resistant (%)	Voriconazole resistant (%)	Echinocandin resistant (%)
*C. albicans*	12	5	5	0	0
*T. ciferrii*	0	0	0	0	0
*C. dubliniensis*	0	0	0	0	0
*N. glabratus*	11.10	11.10	5.50	0.00	0
*M. guilliermondii*	0	0	0	0	0
*C. lusitaniae*	14	0	0	0	0
*C. parapsilosis*	0	6	12	2	0
*C. tropicalis*	0	4	4	0.00	0
Total	3.30	4	4	0	0

## Discussion

We report 6 years of experience with candidemia at a tertiary centre in the DR, including clinical characteristics and susceptibility patterns. The predominant comorbidities in the DR were hypertension, chronic pulmonary disease, and nephropathy. Prior antibiotic use, longer ICU stays, and the necessity for vasopressors and central catheters were also important variables. This aligns with studies in the US showing chronic medical conditions like acute kidney injury and heart disease as risk factors along with exposure to antibiotics [8]. Our multivariable analysis showed that only vasopressor use and greater age were independently associated with mortality, likely reflecting illness severity and established age-related risk. Other comorbidities were not significant after adjustment, possibly due to sample size. These findings underscore the importance of early recognition and targeted management in critically ill and older patients. Mortality was similar to the US at 47% [1, 3].

Differences between prevalent species in the US and the DR exist, with the most common species in the DR reported as *C. tropicalis*, followed by *C. parapsilosis*, *C. albicans*, and *N. glabratus.* This contrasts with epidemiology in the US, where the most common causes are *C. albicans* and species other *Candida spp.* constitute approximately 50% of candidemia [1]. Thus, IDSA guidelines recommend echinocandins as the preferred empiric therapy for coverage of *C. albicans* and the most common species other *C. albicans* [1]. In our study, *C. tropicalis* and *C. parapsilosis* were more common than *C. albicans.* This aligns with recent studies showing a rise in *Candida spp.* other than *C. albicans* globally [5]. There is a paucity of data in the DR and our study represents only one facility. Survival is highly associated with early appropriate antifungal therapy. Thus, understanding local epidemiology and susceptibilities can help local clinicians guide therapy and for the development of local treatment guidelines. Further studies are needed to assess if these trends are present in other facilities across the DR.

Recent global surveillance studies have reported shifts in species distribution and emerging antifungal resistance. Data from the SENTRY program show a decline in *C. albicans* and a rise in *N. glabratus*, *N. glabratus*, *C. parapsilosis*, and *C. tropicalis*, with notable regional differences—*C. tropicalis* being more common in the Asia-Pacific region and *N. glabratus* in North America [5, 9]. Resistance to fluconazole and echinocandins remains uncommon overall but is increasing in *N. glabratus* and *C. tropicalis*, particularly in North America [5, 9]. Our findings align with these global trends and highlight the importance of ongoing local surveillance to guide empiric therapy and inform antimicrobial stewardship in settings with distinct epidemiology like the DR.

Susceptibility profiles for various *Candida* species show differences between the DR and reported global trends. In the DR, *C. albicans* exhibits the highest resistance to amphotericin B at 12% and flucytosine resistance at 7.1%. *C. tropicalis* and *C. parapsilosis* also show significant resistance to flucytosine and fluconazole at rates of 5.8% and 12%, respectively. SENTRY data indicate that *C. albicans* has a lower fluconazole resistance rate (0.3%), *N. glabratus* has an increasing resistance against fluconazole, particularly in North America and the Asia-Pacific regions. Echinocandin and voriconazole resistance remains low both in SENTRY and in our study [5]. Other studies in the Latin America region show resistance rates to fluconazole under 3% [6].

Our study had many limitations, including its single-centre retrospective design. This may limit our ability to generalize this data for other sites in the DR. However, to our knowledge, we report the largest cohort of candidemia and antifungal susceptibility profiles in the DR. We found similarities in risk factors and mortality from candidemia in the DR compared to the US. However, the epidemiology of *Candida* species involved in BSIs and their resistance profiles differ from the US and other countries in Latin America. This may be of use for clinicians and for the development of treatment guidelines. Further studies are needed to characterize susceptibility patterns from a wider geographic distribution in the DR and from different types of facilities.

## Supporting information

10.1017/S0950268825100496.sm001 Rojas-Fermín et al. supplementary material Rojas-Fermín et al. supplementary material

## Data Availability

De-identified data may be shared by the corresponding author upon reasonable request and with appropriate institutional approvals.

## Supplementary material

The supplementary material for this article can be found at http://doi.org/10.1017/S0950268825100496.
