# Supplementary material for: Candidemia in the Dominican Republic: species distribution, resistance, clinical characteristics, and outcomes at a tertiary care hospital
Source: Epidemiol Infect. 2025 Aug 26;153:e98. doi: 10.1017/S0950268825100496 (PMC12415796; doi:10.1017/S0950268825100496)
Supplement: Rojas-Fermín et al. supplementary material [file S0950268825100496sup001.docx]

# Supplementary Tables

## Supplement 1. Univariate Associations Between Clinical Characteristics and Mortality in Patients with Candidemia

| **Variable** | **Unadjusted OR** | **P-value** |
| --- | --- | --- |
| **Sex** | 0.801 | 0.527 |
| **Transfusion** | 1.756 | 0.108 |
| **Immunomodulators** | 2.663 | 0.417 |
| **Chemotherapy** | 0.920 | 1.000 |
| **Solid organ transplant** | 4.135 | 0.045* |
| **Peritoneal dialysis** | 1.408 | 0.736 |
| **Abdominal surgery** | 1.159 | 0.773 |
| **Corticosteroid use** | 1.165 | 0.697 |
| **Antimicrobial use** | 1.318 | 0.827 |
| **Prior hospitalization** | 1.418 | 0.280 |
| **Vasopressors** | 3.943 | 0.000* |
| **Mechanical ventilation** | 2.592 | 0.002* |
| **Pleural drain** | 0.659 | 0.524 |
| **Nasogastric catheter** | 1.897 | 0.037* |
| **Central venous catheter** | 0.993 | 1.000 |
| **Bladder catheter** | 1.563 | 0.181 |
| **Central venous catheter** | 2.571 | 0.004* |
| **Sepsis** | 2.647 | 0.005* |
| **Critical illness** | 3.960 | 0.000* |
| **Age** | 1.017 | 0.001* |

## Supplement 2. Multivariable Logistic Regression Identifying Independent Predictors of Mortality: Acute Clinical Factors

| **Variable** | **Regression Coefficient (Log OR)** | **Standard Error** | | **P-value** | | **95% Confidence Interval** |
| --- | --- | --- | --- | --- | --- | --- |
| **Transfusion** | 0.307 | 0.388 | | 0.427 | | [-0.453, 1.0688] |
| **Solid organ transplant** | 0.974 | 0.760 | | 0.200 | | [-0.5161, 2.4658] |
| **Vasopressors** | 0.912 | 0.347 | | 0.008* | | [0.2305, 1.5938] |
| **Mechanical ventilation** | 0.613 | 0.447 | | 0.170 | | [-0.2628, 1.4894] |
| **Nasogastric tube** | -0.089 | 0.361 | | 0.805 | | [-0.7972, 0.6188] |
| **Bladder catheter** | -0.133 | 0.374 | | 0.722 | | [-0.8671, 0.601] |
| **Central venous catheter** | 0.614 | 0.383 | | 0.108 | | [-0.1361, 1.3654] |
| **Sepsis** | 0.610 | 0.401 | | 0.128 | | [-0.1759, 1.3967] |
| **Critical illness** | 0.866 | 0.471 | | 0.066 | | [-0.0582, 1.7911] |
| **Age** | 0.026 | | 0.006 | | 0.000* | [0.0139, 0.0392] |

**Supplement 3.** Multivariable Logistic Regression Model: Age and Sex as Predictors of Mortality

| **Variable** | **Regression Coefficient (Log OR)** | **Standard Error** | **P-value** | **95% Confidence Interval** |
| --- | --- | --- | --- | --- |
| **Intercept** | -0.624 | 0.302 | 0.038* | [-1.2172, -0.0318] |
| **Age** | 0.017 | 0.005 | 0.001* | [0.0069, 0.0272] |
| **Sex** | -0.321 | 0.296 | 0.278 | [-0.9026, 0.2595] |
